# Book Review: Positive Psychology: The Basics

**DOI:** 10.3389/fpsyg.2021.719489

**Published:** 2021-07-07

**Authors:** Zhiwu Zhang

**Affiliations:** School of Foreign Studies, Changsha University of Science and Technology, Changsha, China

**Keywords:** positive psychology, positive emotions, well-being, optimism, flow, positive relations, grit

Practice-oriented psychology has long earned the reputation of emphasizing on problems, negative emotions, or what needs to be fixed, typical and prominent topics including stress, anxiety and depression. However, with the publication of Seligman and Csikszentmihalyi's (2000) seminal paper at the turn of the twenty-first century, Positive psychology, a new subfield of psychology, walked on to the center stage, shifting its focus to a wider range of new topics: happiness, optimism, hope, well-being, meaning, empathy, resilience, and grit (Lopez and Snyder, 2009). This book, *Positive Psychology: The Basics*, written by Dr. Rona Hart, a veteran in the field of applied positive psychology, comes as a timely summary of the core concepts, theories, and empirical researches after it has undergone over two-decade expansion, offering insights for future development.

The thirteen chapters in this book could be divided into three parts, with Chapter 1 as an introduction of the journey of positive psychology as a discipline, Chapter 13 as an overview of the applied side of positive psychology in life, and Chapters 2–12 covering updated theoretical and empirical development of core concepts, to be specific, happiness (Chapter 2), well-being (Chapter 3), positive emotions and emotional intelligence (Chapter 4), optimism and hope (Chapter 5), goal pursuit and change (Chapter 6), self-regulation and grit (Chapter 7), flow (Chapter 8), meaning in life (Chapter 9), character strengths and virtues (Chapter 10), positive relationships (Chapter 11) and stress, coping, resilience, and post-traumatic growth (Chapter 12). In each chapter, the author usually follows a reader-friendly way of a guidebook, providing definitions to clarify the concepts, elaborating on their importance as well as factors that influence and are influenced by those constructs, reviewing evidence-based benefits and deficiencies, and exploring established leading models.

Chapter 1 presents an overview of positive psychology, including its origins, development, mission, discipline contours, challenges and three prospective future paths. Through this brief and succinct delineation, readers are equipped with a bird's eye view of its past, present and possible future. In Chapter 2, after defining happiness, the author offers a summary of the benefits of happiness based on a review of research findings, then analyzes the determinants of happiness based on the Sustainable Happiness Model, complemented with a wider spectrum of predictors of happiness evidenced by various knowledge sources. Besides, a variety of evidence-based interventions that target to boost happiness are also introduced. In a similar vein, Chapter 3 defines well-being after a conceptual distinction between hedonia and eudaimonia, followed by detailed analysis of well-being models such as Self-determination Theory, PERMA, the Happiness and Meaning Orientation Model. Predictors of well-being and intervention measures are also discussed. Chapter 4 expounds on the Broaden-and-Build Theory of positive emotions and its key functions, together with detailed analysis of leading models of emotional intelligence. Chapter 5 begins by explicating three leading models of optimism and then focuses on empirical findings related to optimism and hope. Chapter 6 first introduces goal theories, centering around the relationship between goals and well-being, then reviews key characteristics of goals and two models of change: the Transitions Model and the Transtheoretical Model. Chapters 7–10 explore briefly about self-regulation and grit, flow, meaning in life, character strengths and virtues, respectively, tapping into their concepts, benefits and upshots. Chapter 11 examines in great length the construct of positive relationships, exploring ingredients that make up beneficial high-quality relationships through a thorough review of theories and empirical findings. Besides, their impact on both physical and psychological well-being are discussed. To overcome the misconception that positive psychology does not address negative emotions, Chapter 12 unpacks four interrelated concepts: stress, coping, resilience and posttraumatic growth. After analyzing how stress occurs and the consequence of stress, the author offers a summary of coping strategies, reviews predictors and outcomes of resilience, and ends with an exploration about models, outcomes and facilitators of post-traumatic growth. Chapter 13 dedicates itself to the applied area of positive psychology and presents an overview of theoretical and empirical studies about positive psychology interventions. A variety of programs, models and positive activities are introduced, together with their optimal conditions and outcomes.

As the first jargon-free introductory book about positive psychology, this book boasts the following merits: firstly, the topics covered are rather encompassing and comprehensive, including key concepts and constructs of this emerging field of positive psychology; within each topic, relevant theories, empirical researches and interventions are well-introduced. Secondly, the breadth and depth of each topic are guaranteed, as the author adopts both longitudinal and cross-sectional exploration about each topic, thus enables readers to gain a clear picture of its past, present, and possible future. What's more, each topic is presented with a rich variety of literature-based views, models, intervention programs, inventories, and questionnaires, providing well-thought stepping stones for practice and research. Lastly, the layout within each chapter is reader-friendly. It is quite easy to identify the key information, the hierarchy and logic within each chapter through the different fonts, sizes, words in italics, bold, and boxed areas.

Overall, through the accessible and comprehensive introduction of this burgeoning branch of psychology, this book provides readers with a fundamental overview of theories, researches and practices within positive psychology. Its rich literature resources and well-established leading models within each topic-centered chapter are springboards for students, researchers and practitioners alike. It serves as a window to this fledgling field and earns itself a must read for both newcomers and veterans.

## Author Contributions

The author confirms being the sole contributor of this work and has approved it for publication.

## Conflict of Interest

The author declares that the research was conducted in the absence of any commercial or financial relationships that could be construed as a potential conflict of interest.
